# Intravascular hemolysis associated with* Candidatus Mycoplasma hematoparvum *in a non-splenectomized dog in the south region of Iran

**Published:** 2014

**Authors:** Hassan Sharifiyazdi, Mohammad Abbaszadeh Hasiri, Amin Hosein Amini

**Affiliations:** *Department of Clinical Studies, School of Veterinary Medicine, Shiraz University, Shiraz, Iran.*

**Keywords:** Dog, Hemoplasma, Hemotropic mycoplasma, *Mycoplasma hematoparvum*

## Abstract

A 2-year-old male Pekingese dog was referred to Shiraz University’s Veterinary Teaching Hospital for anorexia and depression. The case had no history of surgery. Physical examination revealed no abnormalities except mild depression and fever. Small, coccoid, epicellular bacteria were detected on erythrocytes by microscopic examination of the Giemsa-stained blood smears. Abnormalities noted in the complete blood count included regenerative anemia characterized by a marked reticulocytosis. Examination of the plasma showed visual evidence of slight intra vascular hemolysis. In addition, Howell-Jolly bodies, nucleated RBCs, increased immature neutrophils and thrombocytosis were found in this case. The urine was strongly positive for bilirubin, and the urine sediment had abundant bilirubin crystals. For polymerase chain reaction (PCR) purpose, total DNA was extracted from blood sample collected from dog. PCR was positive and phylogenetic analysis of concatenated data showed our isolate clustered within *Candidatus Mycoplasma hematoparvum *group. Treatment was performed by oral ciprofloxacin and prednisolone. The clinical signs improved after three days. Two month follow-up showed no recurrence. In conclusion, hemoplasmosis should be considered as a differential diagnosis in dogs with hemolytic process and pyrexia. The PCR evaluation for hemoplasma DNA should be included in the investigation of such cases to enable the rapid detection of this infection, which may be more common than previously estimated. Besides, ciprofloxacin might have an effect on treatment of hemoplasma in dogs, however, conducting further case studies are necessary to recommend successful treatment.

## Introduction

There are two species of hemotropic mycoplasma (also called hemoplasma) that infect dogs which are known so far: *Mycoplasma hemocanis* and* Candidatus Mycoplasma hematoparvum *(*CMhp*)*.*^1^^,^^2^ The main form of transmission is probably through blood sucking arthropods such as the tick *Rhipicephalus sanguineus* whose main geographical distribution is associated with the Mediterranean and sub-Mediterranean climates.^3^ These extracellular parasites attach to the surface of canine erythrocytes, causing hemolytic anemia mostly through extravascular destruction of erythrocytes by the mononuclear phagocyte system.^2^ Infection with these hemoplasmas generally only induces clinically significant anemia in splenectomized or immunocompromised dogs, although latent infections may cause subclinical anemia.^2^^,^^4^ *Candidatus M. hematoparvum *was first described in association with anemia in a splenectomized dog undergoing chemotherapy for leukaemia.^5^ Most non-splenectomized dogs infected with hemoplasma do not develop clinical evidence of disease and do not have sufficient number of organisms present in the blood to be recognized during routine blood film examinations. Besides, diagnosis of these pathogens by serological responses can be unspecific. Therefore, molecular techniques that are simpler, faster, less hazardous and usually more sensitive have been developed for hemoplasmas species detection.^2^^,^^6^

The present case report describes the first report of hemolytic *Mycoplasma* infection in the south of Iran and hemolytic disorders in a non-splenectomized dog infected with *CMhp.*

## Case Description

A 2-year-old male Pekingese dog was referred to Veterinary Teaching Hospital of Shiraz University for anorexia and depression. The case had no history of surgery. Body temperature was 40 ˚C, heart rate (130 beat per min), respiratory rate (20 breath per min) and body condition was normal. Physical examination revealed no abnormalities except mild depression. 

Hematological examination was carried out using automatic cell counter (Exigo, Stockholm, Sweden) and blood smears were prepared for Giemsa staining.

## Results

**Fig. 1:**
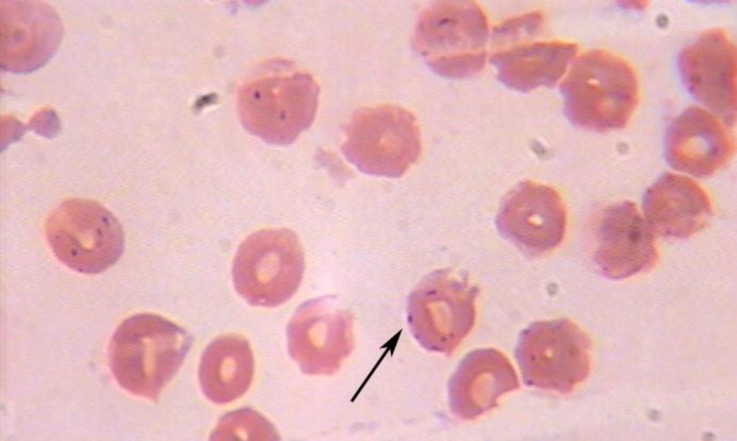
Coccoid bacteria were detected on the surface of erythrocytes of the affected dog, (Giemsa, 1000×).

Small, coccoid, epicellular bacteria were detected on erythrocytes by microscopic examination of the Giemsa-stained blood smears (Fig. 1). 

Abnormalities noted in complete blood count included regenerative anemia characterized by a marked reticulo-cytosis (278 × 10^9^ L^-1^) and packed cell volume of 50.0%. Examination of the plasma showed visual evidence of slight intravascular hemolysis (hemoglobinemia). In addition, Howell-Jolly bodies, nucleated RBCs (4 per 100 WBC), increased immature neutrophils (Band form; 0.36 × 10^9^ L^-1^, reference interval: 0 to 0.30 ×10^9^ L^-1^) and thrombocytosis (705 × 10^9^ L^-1^, reference interval: 211 to 621 × 10^11^ L^-1^) were found in this case. 

**Fig. 2:**
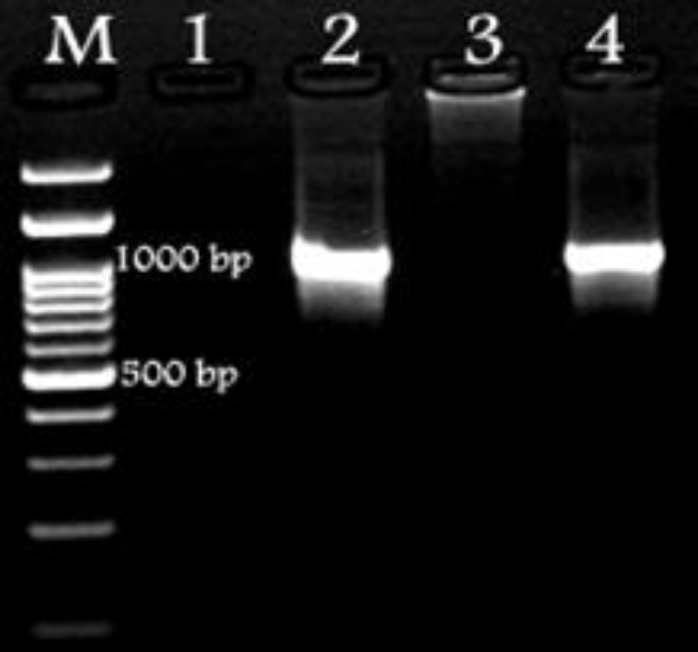
PCR amplification provided from canine sample (Lane 4) compared with the molecular weight marker (Lane M: 100 bp) and positive control (Lane 2). Distilled water (Lane 1) and DNA from a healthy dog (Lane 3) are negative controls

The urine was strongly positive for bilirubin, and the urine sediment had abundant bilirubin crystals. For polymerase chain reaction (PCR) purpose, total DNA was extracted from blood sample collected from dog using the DNeasy^®^ Blood and Tissue Kits (Qiagen, Hilden, Germany) according to the manufacturer’s instructions. Amplification of the 16S rDNA was performed using the lyophilized PCR micro tubes (Model Accupower PCR PreMix; BioNeer Co., Seoul, Korea) and oligonucleotides targeting 16S rDNA regions specific for HM (16S_ HEMO forw: GGCCCATATTCCT (AG) CGGGAAG; 16S_ HEMOrev: AC (AG) GGATTACTAGTGATTCCA).^7 ^The amplified products were sequenced directly using the capillary DNA analyzer (Model ABI 3730; Applied Biosystems, Foster City, USA). The 16S rDNA sequence obtained was compared to GenBank entries using the BLAST tool provided by National Center for Biotechnology Information (NCBI).^8^ Different related *Mycoplasma* species were used for phylogenetic analysis. Multiple sequence alignments and construction of a phylogenetic tree were made with the neighbor-joining method using the software, MEGA (Version 4.0; Biodesign Institute, Tempe, USA). ^9^ PCR was positive by producing a specific fragment of ~1000 bp from DNA of the blood as shown in Figure 2.

**Fig. 3:**
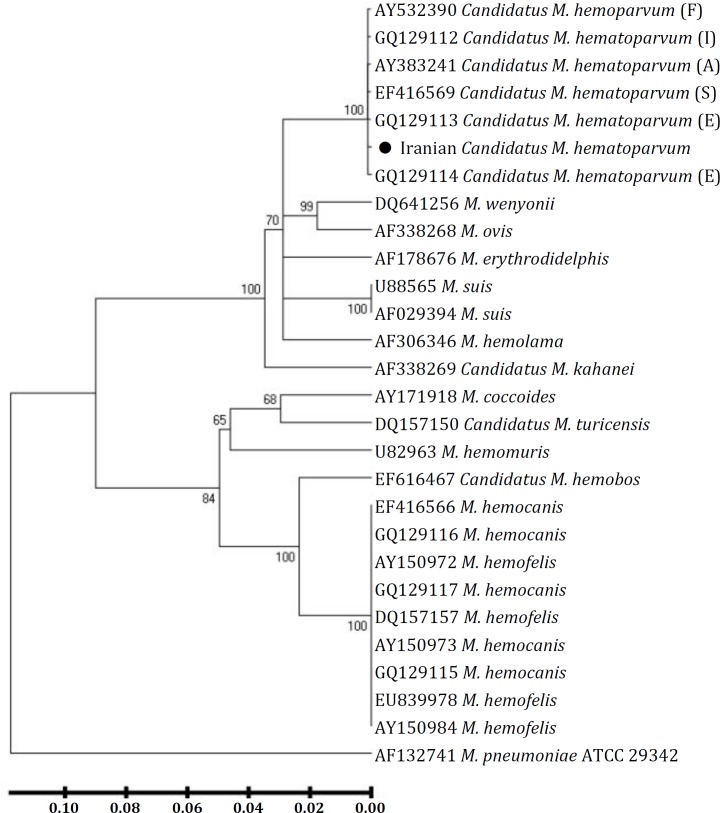
Phylogenetic analysis using partial sequences of 16S rDNA showing the position of the sequenced canine hemoplasma isolates from Iran among the hemotropic mycoplasma group. Numbers in the relevant branches refer to the values of boot-strap probability of 1,000 replications. *M*.= *Mycoplasma*. S= Switzerland; F= France I= Italy, E= Spain and A= USA. The phylogenetic tree was constructed using the neighbor-joining method. A nucleotide sequence of the 16S rRNA gene of *M. pneumoniae* ATCC 29342 with accession number AF132741 was included as an out-group.

Phylogenetic analysis of concatenated data showed our isolate clustered within the *CMhp *group (Fig. 3). Furthermore, comparative sequence analysis using the obtained 16S rDNA sequence (Accession number KC 762746) demonstrated the highest homology (more than 99.0%) to *CMhp*, previously described by Novacco *et* *al*.^ 10 ^(Accession numbers GQ129112, GQ129113: Italy) and Wengi *et* *al*. 3 (Accession number EF416569: Switzerland). The difference between Iranian sequence and these isolates was only a transition mutation of cytosine (C) to thymine (T) at position 902 based on the accession number KC762746. Also, comparison of an available sequence for *CMhp* (Accession number AY532390) originating from France with Iranian sequence showed the greatest difference (0.7%; 7 nucleotides). 

Treatment was performed by oral ciprofloxacin (Farabi Pharmaceutical Co. Isfahan, Iran; 20 mg kg^-1^, q24h for 7 days) and prednisolone (Aburaihan Co., Tehran, Iran; 0.5 mg kg^-1^, q12h for 3 days). The clinical signs improved after three days. Two month follow-up showed no recurrence. 

## Discussion

This is the first case of canine infection with *CMhp* in the southern region of Iran. This organism, named *CMhp *is smaller than *M. hemocanis* and does not form chains on the erythrocyte surface of dogs.^5^ *Candidatus M. hematoparvum* is genetically more closely related to *Candidatus M. hemominutum* than to *M. hemofelis*, and was isolated from the blood of a splenectomized dog with hematopoietic neoplasia.^5^ The infection has been confirmed by methods of molecular biology and specific PCR is the gold standard for detection of these infections.^12^^,^^13^

Santos showed twenty (11.3%) out of 176 dogs living in rural areas were positive for hemoplasmas, whereas 6 of 104 (5.8%) dogs from urban areas harbored the organism.^13^ Our case lived indoors in an urban area. Blood samples from 460 dogs living in the south of France showed 9.6% were infected with *CMhp *whereas only 3.3% were infected with *M. hemocanis* and 2.6% were infected with both organisms.^14^ However, in Switzerland only 1.2% of dogs had positive real-time PCR results for the canine hemoplasmas. The prevalence in Europe is higher in the Mediterranean countries. It is postulated that the presence or absence of an appropriate vector for transmission of the organisms may explain these differences.^2^

Clinical cases of canine hemoplasmosis have occasionally been reported, but co-factors such as splenectomy or immunosuppression seem to play a role in pathogenesis of the disease. Rare cases of acute disease have occurred in dogs with intact spleen in which no evidence for immunosuppression was found.^2^ However, additional immunological assays (not routinely available) were necessary to better characterize the underlying immunosuppressive conditions. In this case, hemolysis and fever existed in non-splenectomized dog. So it could be postulated that in intravascular hemolysis, hemoplasmas must be considered in differential diagnosis as a suspected causative agent. 

On the other hand, most non-splenectomized dogs infected with hemoplasmas do not have sufficient numbers of organisms present in the blood to be recognized during routine blood film examinations and due to unspecific serological examination, PCR could be recommended. In the present case, we demonstrated this hemoplasma species in a hemolytic dog with regenerative response. 

As mentioned above, a greater regenerative response occurs in hemolytic anemia than in other anemia because the iron and protein of the destroyed RBCs are readily available for erythropoiesis. The polychromasia, anisocytosis, Howell-Jolly bodies, and nucleated RBCs are consistent with regenerative anemia.^15^ In spite of normal PCV (50.0%), ongoing slight intravascular hemolysis was confirmed with light pink to red discoloration of plasma and remarkable regenerative response. Hemoglobinuria was not detected in this case because hemoglobinemia occurred below the threshold for urinary excretion of hemoglobin (> 150 mg dL^-1^). 

Recent research indicated that treatment of *M. hemofelis* infected cats with the fluoroquinolone may offer more effective long-term clearance of organisms than doxycycline.^2^ Orally administered tetracyclines are reported to be effective in treating *M. hemocanis* infections but data are not available on the efficacy of treatment of *CMhp *infection in dogs.^2^ We translated successful cat treatment for this case. The clinical signs improved within three days of initiating treatment and follow-up showed no regression. 

This case was unique in several ways: first in describing *CMhp *infection using sequencing method in Iran. Since ticks and mites as important sources of *Hemobartonella* infection are frequent in this area, it seems this infection may be common in these dogs and all ticks should be removed from the environment; second, in associating hemoplasma infection with pyrexia and intravascular hemolysis in a non-splenectomized dog, and third, successful treatment. 

In conclusion, hemoplasmosis should be considered as a differential diagnosis in dogs with hemolytic process and pyrexia. The PCR evaluation for hemoplasma DNA should be included in the investigation of such cases to enable the rapid detection of this infection, which may be more common than previously estimated. Besides, ciprofloxacin might have an effect on treatment of hemoplasma in dogs, however, conducting further case studies are necessary to recommend successful treatment. 
